# NO Better Way to Protect the Heart during Ischemia–Reperfusion: To be in the Right Place at the Right Time

**DOI:** 10.3389/fped.2015.00006

**Published:** 2015-02-06

**Authors:** Charlotte Farah, Cyril Reboul

**Affiliations:** ^1^EA4278, LaPEC, Université d’Avignon, Avignon, France; ^2^UMR-CNRS 9214, INSERM U1046, Université de Montpellier, Montpellier, France

**Keywords:** ischemia reperfusion, nitric oxide, S-nitrosylation, nitrite, pre-conditioning, post-conditioning

## Introduction

Acute myocardial infarction (MI) is one of the leading causes of mortality worldwide. MI is the heart muscle irreversible death secondary to prolonged ischemia. Over the last few decades, medical progress in how and when to restore blood flow to the ischemic area have markedly improved patient survival. Although early heart reperfusion is acknowledged to be the most effective way to limit infarct size, post-ischemic reperfusion is associated with detrimental effects, such as myocardial stunning, ventricular arrhythmias, microvascular dysfunction, and cell death. The molecular mechanisms of these reperfusion injuries remain to be elucidated and their management is very challenging.

Among the various therapeutic molecular approaches proposed by experimental studies, nitric oxide (NO) role in protecting heart against MI and reperfusion injuries has been widely assessed and discussed (1–4). NO is a gasotransmitter that is abundantly produced in the cardiovascular system mainly by the NO synthase (NOS) enzymes system. Two isoforms, endothelial NOS (eNOS) and neuronal NOS (nNOS), are constitutively expressed in both myocardium and vessels, whereas inducible NOS (iNOS) is detected only in pathological conditions, such as inflammatory and/or oxidative stress. Both eNOS and nNOS are low-NO output Ca^2+^-dependent enzymes, while iNOS is a high-NO output Ca^2+^-independent enzyme. In physiological conditions, NOS form homodimers (“coupled” NOS) that catalyze NO production from l-arginine and O_2_ through electron transfer from NADPH on the reductase domain of one monomer to the oxidase domain of the second monomer. In pathological conditions, such as in the absence of the essential cofactor tetrahydrobiopterin (BH_4_), eNOS can be “uncoupled” to produce O_2_^−^ instead of NO.

In stress conditions, NO protects tissues through two distinct pathways. In the first one, NO activates the soluble guanylate cyclase (sGC) that initiates cyclic guanosine monophosphate (cGMP) production, leading to the activation of protein kinase G (PKG). As sGC is the major cell receptor for NO and the NO/sGC/cGMP/PKG pathway plays a critical role in both myocardium excitation–contraction coupling and cardiovascular function regulation (5–8), NO cardioprotective role was first attributed to PKG activation (9–11). However, a second pathway in which proteins are directly modified by NO addition to sulfhydryl residues, a process known as S-nitrosylation (SNO), has recently emerged in the scientific literature. Although PKG activation pathway has been largely involved in NO-mediated cardioprotection (11–13), SNO is now taking the front stage and is considered to be a key player in cardioprotection through (i) the transient modification of protein activity and/or (ii) their protection from irreversible oxidation (14–17). Indeed, Sun et al. (18) showed that reduced heart vulnerability to ischemia–reperfusion (IR) following acute ischemic preconditioning is mainly related to SNO signaling and not to PKG activation through the NO–SGC–cGMP pathway. Accordingly, we found that in exercise training-induced cardioprotection against IR injuries, protein SNO level, but not cGMP level, increased during early reperfusion (19). The same year, Methner et al. (20), using a Cre/loxP approach to selectively ablate type I PKG in cardiomyocytes, demonstrated that ischemic post-conditioning reduced infarct size in these mice like in wild type controls. Moreover, they showed that the cardioprotective effect against IR injury of mitochondria-targeted *S*-nitrosothiol (MitoSNO), which allows NO and *S*-nitrosothiol accumulation in mitochondria, was comparable in mice that specifically lack PKG in cardiomyocytes and in controls. This indicates that MitoSNO cardioprotective effect is independent of PKG.

The current literature strongly supports NO implication in cardioprotection. However, the mechanism is still debated and whether increased NO availability during IR is cytoprotective remains to be demonstrated. Here, we discuss how NO might contribute to protect heart and particularly the importance of NO (i) localization, (ii) concentration, and (iii) time of availability during IR.

## Right Place

NO signaling depends on NOS cardiomyocyte subcellular localization (8, 21). Indeed, eNOS-catalyzed NO production occurs almost everywhere in the cell, thus leading to SNO of many targets (membrane and cytosolic proteins). On the other hand, nNOS colocalizes with SERCA and RyR and is associated with SNO mainly of reticulum sarcoplasmic proteins (16, 22). Although more than 1000 proteins are *S*-nitrosylated in hearts, recent findings suggest that cardiac mitochondrial proteins are key targets of NO-dependent cardioprotection. For instance, Sun et al. (23) reported that during ischemic preconditioning, caveolae-mediated transduction of eNOS/NO/SNO signaling to end-effector mitochondria leads to cardioprotection. Recently, Chouchani et al. (24) elegantly demonstrated that MitoSNO-induced heart protection could be explained by SNO of the ND3 subunit of mitochondrial complex I (on Cys39) to transiently lock it in a low-activity state, thus decreasing ROS production during early reperfusion. This SNO of the ND3 subunit was observed using an ischemic preconditioning experimental approach. Thus, through SNO, NO could directly protect mitochondrial proteins during IR. However, NO has also indirect effects. Indeed, Huang et al. (25) reported that SNO of G-protein-coupled receptor kinase 2 (GRK2) is the primary target of NO cardioprotective activity. Although GRK2 is a cytosolic protein that regulates the activity of G-protein coupled receptors, the authors found that, during IR, GRK2 translocates to mitochondria and activates the cell death pathway. SNO-dependent inhibition of GRK2 could then have a protective effect, mainly by preserving mitochondrial integrity.

S-nitrosylation clearly has beneficial effects during IR when targeting specifically mitochondria; however, incorporation of a NO moiety (NO^+^ or NO) to a sulfur atom can also be deleterious. Indeed, hypernitrosylation of RyR2 channels via the TNF-α/caspase 8 pathway increases Ca^2+^ leak from the sarcoplasmic reticulum (SR), thus contributing to cell death during IR (26). In this work, reduction of RyR2-SNO by using a caspase 8 inhibitor (Q-LETD-OPh) reduced infarct size in mice. On the other hand, RyR2 hyponitrosylation also promotes SR Ca^2+^ leak and thus might increase heart vulnerability to IR (27). Based on these results, Beigi et al. (28) demonstrated that dynamic SNO/denitrosylation reactions are essential for cardiovascular homeostasis.

Taken together, these data clearly show that SNO plays a key role in cardioprotection and also illustrate that this positive effect depends on the target and thus on the subcellular localization of NO synthesis. NO concentration could also be important and seems to be directly linked to the cellular redox state during IR (4). Indeed, although NO has undoubtedly a cardioprotective function, it could go over to the dark side, particularly if too much NO is produced or if it is available at the wrong moment.

## Right Concentration

In pro-oxidative conditions, such as during myocardial IR, NO can react with O_2_^−^ to form peroxynitrite (ONOO^−^), a cytotoxic molecule that is considered to be the main trigger of reperfusion injuries (29). Consequently, it is debated whether increasing NO bioavailability during IR has a cardioprotective effect (30). For instance, NOS overexpression, BH_4_ supplementation, or nitrite supplementation reduces heart sensitivity to IR (31–34). Also, numerous studies showed the cytoprotective function of iNOS during the specific late phase of preconditioning (35). But conversely, Csonka et al. (36) demonstrated that classical preconditioning induces cardioprotection by reducing NO harmful accumulation during early reperfusion. Consistently, the group of Zweier suggested that, in a pro-oxidative environment, transient limitation of NO synthesis via temporary S-gluthationylation-mediated NOS uncoupling might exert a cardioprotective effect by limiting ONOO^−^ overproduction (37, 38). Similarly, we demonstrated that exercise-induced cardioprotection requires eNOS uncoupling during early reperfusion to limit excessive NO production and thus to reduce nitro-oxidative stress (19). If eNOS uncoupling during IR is corrected by BH_4_ supplementation, higher ONOO^−^ production and increased infarct size are observed (19). Nonetheless, in this rat model of cardioprotection, reduced NO synthesis due to eNOS uncoupling during early reperfusion was counterbalanced by increased storage of NO metabolites (nitrite and *S*-nitrosothiols) in hearts of exercised rats (compared to sedentary controls). This allowed maintaining a high level of protein SNO, and thus of cardioprotection, during early reperfusion.

NO availability could mostly explain the discrepant findings concerning the effects of increasing or reducing NO synthesis during IR.

## Right Time

The time of NO availability for SNO of key proteins during IR seems to be critical. Recently, Chouchani et al. (24) showed that SNO of mitochondrial complex I by MitoSNO can be observed only after ischemia and does not occur in normoxic hearts. The authors suggested that ischemia allows the exposure of Cys39 in the ND3 subunit of complex I for SNO. Considering that this mechanism is a key point of SNO-dependent cardioprotection, we can hypothesize that any strategy to make NO available during the ischemic period could contribute to protect the myocardium during IR. However, during ischemia, eNOS is not functional because it is monomerized, its activation by phosphorylation blunted (19) and its substrates (especially O_2_) are lacking. Therefore, NO availability in ischemic conditions is mainly dependent on NO metabolites (i.e., nitrite and *S*-nitrosothiols). Nitrite, a NO reservoir, is reduced to NO during ischemia by nitrite reductase (32) and *S*-nitrosothiols can decompose to liberate NO (39). Thus, oral treatment with nitrite (40) or acute nitrite administration before ischemia (41) increases NO metabolite storage, providing cardioprotection against IR. Moreover, nitrite therapy mediates cardioprotection by modulating mitochondrial function, leading to increased SNO of the ND3 subunit of the mitochondrial complex I (24). We (19) and others (42), showed that the beneficial effects of exercise on IR injuries are mainly mediated through the increase of the NO metabolite reserve before ischemia. In our work, such increase was associated with higher protein SNO level during early reperfusion. Finally, Calvert et al. (42) clearly demonstrated, using mice in which β3-adrenergic receptor (which plays a key role in eNOS activation) was knocked out that inhibition of exercise-induced NO metabolite storage increase abolishes the cardioprotective effects of exercise against IR.

Altogether, these results indicate that ensuring NO availability during the ischemic period by increasing NO metabolites storage and improving SNO during early reperfusion are key steps for efficient cardioprotection.

## Conclusion

NO is essential to limit the deleterious effects of post-ischemic reperfusion. Experimental studies indicate that NO exerts its cardioprotective effect against ischemia–reperfusion injuries not only directly through protein SNO but also indirectly through the NO/GCs/GMPc/PKG signaling pathway. Among the many cellular targets of NO, SNO of mitochondrial proteins during IR seems to play a key role. However, the efficiency of NO-induced cardioprotection depends on (i) its concentration, because NO can also generate nitro-oxidative stress (through the formation of ONOO^−^), (ii) its subcellular localization, and (iii) when it is available. Thus, improving NO metabolite (nitrites and *S*-nitrosothiols) storage before and during ischemia, to optimize NO availability and SNO during ischemia and early reperfusion, is an efficient strategy. Nevertheless, further studies are needed to better understand NO metabolism dynamics during IR in order to liberate the right amount of NO in the right place and at the right time.

## Conflict of Interest Statement

The authors declare that the research was conducted in the absence of any commercial or financial relationships that could be construed as a potential conflict of interest.
